# Variants in Pharmacokinetic Transporters and Glycemic Response to Metformin: A Metgen Meta‐Analysis

**DOI:** 10.1002/cpt.567

**Published:** 2017-02-03

**Authors:** T Dujic, K Zhou, SW Yee, N van Leeuwen, CE de Keyser, M Javorský, S Goswami, L Zaharenko, MM Hougaard Christensen, M Out, R Tavendale, M Kubo, MM Hedderson, AA van der Heijden, L Klimčáková, V Pirags, A Kooy, K Brøsen, J Klovins, S Semiz, I Tkáč, BH Stricker, CNA Palmer, LM 't Hart, KM Giacomini, ER Pearson

**Affiliations:** ^1^ Department of Biochemistry and Clinical AnalysisFaculty of Pharmacy, University of SarajevoSarajevoBosnia and Herzegovina; ^2^ Division of Molecular and Clinical MedicineSchool of Medicine, University of DundeeDundeeUK; ^3^ Department of Bioengineering and Therapeutic SciencesUniversity of California, San FranciscoSan FranciscoCaliforniaUSA; ^4^ Department of Molecular Cell BiologyLeiden University Medical CenterLeidenThe Netherlands; ^5^ Department of EpidemiologyErasmus Medical CenterRotterdamThe Netherlands; ^6^ Inspectorate of HealthcareUtrechtThe Netherlands; ^7^ Department of Internal Medicine 4Faculty of Medicine, Šafárik UniversityKošiceSlovakia; ^8^ Pasteur University HospitalKošiceSlovakia; ^9^ Latvian Biomedical Research and Study CentreRigaLatvia; ^10^ Department of Clinical Chemistry and PharmacologyOdense University HospitalOdenseDenmark; ^11^ Treant ZorggroepLocation BethesdaHoogeveenThe Netherlands; ^12^ Bethesda Diabetes Research CentreHoogeveenThe Netherlands; ^13^ Core for Genomic MedicineRIKEN Center for Integrative Medical SciencesYokohamaJapan; ^14^ Division of ResearchKaiser Permanente Northern CaliforniaOaklandCaliforniaUSA; ^15^ Department of General PracticeEMGO+ Institute for Health and Care Research, VU University Medical CenterAmsterdamThe Netherlands; ^16^ Department of Medical BiologyFaculty of Medicine, Šafárik UniversityKošiceSlovakia; ^17^ Department of Public HealthClinical Pharmacology and Pharmacy, University of Southern DenmarkOdenseDenmark; ^18^ International University of SarajevoFaculty of Engineering and Natural SciencesSarajevoBosnia and Herzegovina; ^19^ Department of Internal MedicineErasmus Medical CenterRotterdamThe Netherlands; ^20^ Department of Molecular EpidemiologyLeiden University Medical CenterLeidenThe Netherlands; ^21^ Department of Epidemiology and BiostatisticsEMGO+ Institute for Health and Care Research, VU University Medical CenterAmsterdamThe Netherlands; ^22^ Institute for Human GeneticsUniversity of California, San FranciscoSan FranciscoCaliforniaUSA

## Abstract

Therapeutic response to metformin, a first‐line drug for type 2 diabetes (T2D), is highly variable, in part likely due to genetic factors. To date, metformin pharmacogenetic studies have mainly focused on the impact of variants in metformin transporter genes, with inconsistent results. To clarify the significance of these variants in glycemic response to metformin in T2D, we performed a large‐scale meta‐analysis across the cohorts of the Metformin Genetics Consortium (MetGen). Nine candidate polymorphisms in five transporter genes (organic cation transporter [OCT]1, OCT2, multidrug and toxin extrusion transporter [MATE]1, MATE2‐K, and OCTN1) were analyzed in up to 7,968 individuals. None of the variants showed a significant effect on metformin response in the primary analysis, or in the exploratory secondary analyses, when patients were stratified according to possible confounding genotypes or prescribed a daily dose of metformin. Our results suggest that candidate transporter gene variants have little contribution to variability in glycemic response to metformin in T2D.

**Study Highlights**

**WHAT IS THE CURRENT KNOWLEDGE ON THE TOPIC?**

☑ Published studies on the impact of the polymorphisms in organic cation transporter genes on metformin response have been inconsistent and mostly hindered by small sample size.

**WHAT QUESTION DID THIS STUDY ADDRESS?**

☑ This study explored whether variations in metformin transporter genes affect glycemic response to metformin through a large‐scale meta‐analysis.

**WHAT THIS STUDY ADDS TO OUR KNOWLEDGE**

☑ Nine candidate variants in membrane transporter genes showed no significant effect on metformin response, assessed as HbA_1c_ reduction, in patients with T2D.

**HOW THIS MIGHT CHANGE CLINICAL PHARMACOLOGY OR TRANSLATIONAL SCIENCE**

☑ Candidate variants in transporter genes, despite their role in metformin pharmacokinetics, showed no relevant contribution to variability in metformin efficacy and might not be useful as predictors for individualized treatment with metformin.

Metformin is the first‐line pharmacological therapy for type 2 diabetes (T2D) and the most widely prescribed antidiabetic drug. The glycemic response to metformin is, however, highly variable. In patients receiving metformin as an initial treatment for T2D, less than two‐thirds achieve acceptable glycemic control or a target HbA_1c_ <7% (53 mmol/mol).^1^, ^2^ Genetic factors play an important role in the variable glycemic response to metformin, with up to 34% of variance in HbA_1c_ reduction explained by common genetic variants, each conferring a small to moderate impact.^3^ As a result, a large sample size is required for pharmacogenetic studies aiming to discover these common metformin response variants.

Previously published studies of metformin pharmacogenetics have mostly focused on the candidate genes involved in drug pharmacokinetics, with the expectation that these might have a large clinical effect. For example, polymorphisms in the transporters, organic anion‐transporting polypeptide 1B1 and breast cancer resistance protein, have been associated with large effect sizes with the pharmacokinetics and pharmacodynamics of several drugs, including statins.^4^ Metformin is not metabolized and is excreted unchanged by the kidneys. Its primary mode of action seems to be an increase of hepatic insulin sensitivity through inhibition of gluconeogenesis,^5^ although there is increasing recognition of its role in the gut.^6^ As metformin is an organic cation at physiologic pH, cation‐selective carrier proteins mediate its transport across cell membranes in the intestine, liver, and kidneys. Organic cation transporter 1 (OCT1; encoded by *SLC22A1*) is expressed on the sinusoidal membrane of hepatocytes and is the main transporter of metformin into the liver.^7^ Organic cation transporter 2 (OCT2; encoded by *SLC22A2*) is expressed primarily at the basolateral membrane in the kidney tubular cells and facilitates the uptake of metformin from the blood into the kidneys.^8^ The multidrug and toxin extrusion transporter 1 (MATE1; encoded by *SLC47A1*) and MATE2‐K (encoded by *SLC47A2*), are H^+^/drug antiporters located in the apical membrane of the renal tubular cells, and facilitate metformin excretion from tubular cells into urine.^9^, ^10^, ^11^ A recent study showed that metformin is also a substrate of carnitine/cation transporter 1 (OCTN1; encoded by *SLC22A4*).^12^ OCTN1 is highly expressed at the apical membranes in renal proximal tubules and could also contribute to metformin elimination.^13^, ^14^ To date, several polymorphisms in these transporter genes have been associated with the pharmacokinetics and pharmacodynamics of metformin in healthy volunteers^15^, ^16^, ^17^, ^18^ and with metformin response in T2D.^18^, ^19^, ^20^, ^21^, ^22^, ^23^, ^24^, ^25^ In addition, a few studies have reported gene‐gene interactions between polymorphisms in transporter genes.^18^, ^22^, ^26^ However, the results of these studies have been inconsistent and the impact of the established pharmacokinetic variants on metformin clinical response in T2D is uncertain.^27^ Apart from the different measures of glycemic response used in these studies, the small sample size and reporting bias is another potential explanation for the observed inconsistency.

To clarify the role of genetic variants in these transporters on metformin clinical response, we performed a large‐scale meta‐analysis of the impact of known candidate variants on metformin efficacy in T2D, across the cohorts of recently established Metformin Genetics Consortium (MetGen). This resource now has in excess of 10,000 individuals in whom metformin response can be defined, and offers a unique opportunity for a highly powered pharmacogenetic meta‐analysis of glycemic response to metformin.

## RESULTS

**Figure 1:**
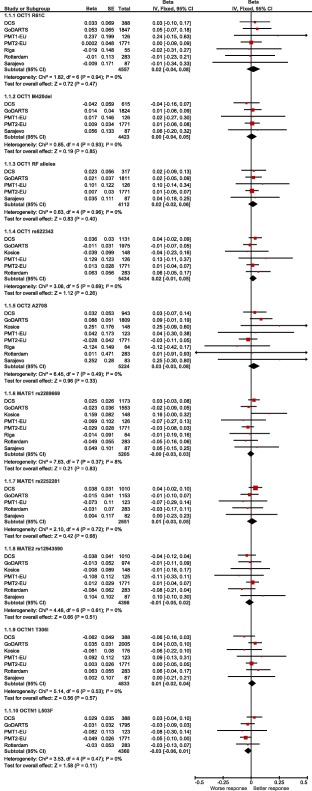
Effects of candidate variants in transporter genes on metformin glycemic response assessed as HbA_1c_ reduction in patients on metformin monotherapy. Beta values obtained from individual studies are presented with 95% confidence interval (CI); arrowheads indicate the CI exceeding the limits of the graph. Overall betas are presented as black diamonds. The organic cation transporter 1 (OCT1) reduced‐function (RF) alleles denote combined genotype for R61C and M420del – number of RF alleles. MATE1, multidrug and toxin extrusion transporter 1. [Color figure can be viewed at wileyonlinelibrary.com]

We studied the effect of nine candidate variants in transporter genes OCT1,^15^, ^16^, ^19^, ^20^, ^23^, ^28^ OCT2,^17^, ^23^, ^25^, ^29^, ^30^ MATE1,^18^, ^21^, ^23^, ^25^, ^28^, ^31^ MATE2‐K,^18^, ^24^ and OCTN1^14^, ^28^ (**Table** 1) on metformin glycemic response in 7,968 MetGen participants of European ancestry. Of these, the definition of metformin response could be aligned for a meta‐analysis in 7,656 participants, of whom 5,836 were initiated on metformin monotherapy and 1,820 were initiated on metformin as add‐on treatment for sulfonylureas (dual therapy; **Table** 2). Forest plots for the meta‐analyses of the individual variants in the monotherapy group are presented in **Figure** 1. There was no significant heterogeneity between the studies for any polymorphism. None of the variants was significantly associated with glycemic response to metformin (**Figure** 1). Similarly, when patients on monotherapy and dual therapy were analyzed together, no single‐nucleotide polymorphism (SNP) showed significant association with HbA_1c_ reduction (**Supplementary Table S1** online). The results from the HOME and SDDS studies, two MetGen cohorts in which metformin was added to insulin therapy, did not show significant impact on metformin response assessed as HbA_1c_ reduction (**Supplementary Table S2** online).

**Table 1:** Single‐nucleotide polymorphisms explored in the meta‐analysis

Gene	dbSNP ID	Nucleotide change	Amino acid change	MAF^a^	Reference
OCT1 (*SLC22A1*)	rs12208357	c.181C>**T**	R61C	0.06	Shu *et al*., 2007^15^; Shu *et al*., 2008^16^; Tzvetkov *et al*., 2009^28^; Zhou *et al*., 2009^19^; Christensen *et al*., 2011^23^
	rs72552763	c.1260GAT>**del**	M420del	0.19^15^, ^45^	Shu *et al*., 2007^15^; Shu *et al*., 2008^16^; Tzvetkov *et al*., 2009^28^; Zhou *et al*., 2009^19^; Christensen *et al*., 2011^23^
	rs622342	Intron A>**C**		0.38	Becker *et al*., 2009^20^; Christensen *et al*., 2011^23^
OCT2 (*SLC22A2*)	rs316019	c.808G>**T**	A270S	0.11	Song *et al*., 2008^29^; Wang *et al*., 2008^30^; Chen *et al*., 2009^17^; Christensen *et al*., 2011^23^; Tkáč *et al*., 2013^25^
MATE1 (*SLC47A1*)	rs2289669	Intron G>**A**		0.42	Becker *et al*., 2009^21^; Tzvetkov *et al*., 2009^28^; Jablonski *et al*., 2010^31^; Christensen *et al*., 2011^23^; Tkáč *et al*., 2013^25^
	rs2252281	g.‐66T>**C**		0.41	Stocker *et al*., 2013^18^
MATE2‐K (*SLC47A2*)	rs12943590	g.‐130G>**A**		0.27	Choi *et al*., 2011^24^; Stocker *et al*., 2013^18^
OCTN1 (*SLC22A4*)	rs272893	c.917C>**T**	T306I	0.41	Yoon *et al*., 2013^14^
	rs1050152	c.1507C>**T**	L503F	0.39	Tzvetkov *et al*., 2009^28^

Minor alleles are shown in bold. dbSNP, single nucleotide polymorphism database; ID, identification; MAF, minor allele frequency; MATE1, multidrug and toxin extrusion transporter; OCT1, organic cation transporter.

a Minor allele frequencies from 1000 Genome Project Phase 3 EUR population (www.ncbi.nlm.nih.gov/projects/SNP).

**Table 2:** Characteristics of cohort participants included in the meta‐analysis

Characteristic	DCS	GoDARTS	Kosice	PMT1‐EU	PMT2‐EU	Riga	Rotterdam	Sarajevo
No. of participants	1,380	3,170	148	125	2,358	64	323	87
Age, years	62.1 ± 10.6	61.9 ± 11.1	57.5 ± 10.4	57.2 ± 12.9	66.2 ± 9.8	59.7 ± 10.7	69.7 ± 10.6	58.2 ± 9.0
Sex, male, %	757 (55)	1,813 (57)	72 (49)	61 (49)	1,283 (54)	19 (30)	147 (46)	37 (43)
BMI, kg/m^2^	30.9 ± 5.2	31.7 ± 5.9	31.5 ± 4.6	37.7 ± 9.1	32.3 ± 6.8	34.4 ± 5.2	29.5 ± 4.7	31.2 ± 4.3
Pretreatment HbA_1c_, %	7.3 ± 1.3	8.9 ± 1.4	7.6 ± 1.1	7.8 ± 1.3	7.8 ± 1.5	7.1 ± 1.1	7.8 ± 1.3	7.8 ± 1.4
On‐treatment HbA_1c_	6.6 ± 0.8	7.2 ± 1.1	7.0 ± 0.8	6.7 ± 0.8	6.5 ± 0.8	6.4 ± 0.5	6.7 ± 0.7	6.7 ± 0.7
Creatinine clearance, ml/min^a^	105 ± 38	93.2 ± 35.4	104 ± 36	80.2 ± 20.1	100.0 ± 38.1	122.4 ± 44.5	85.2 ± 32.6	109.5 ± 30.4
Metformin daily dose, mg	1,089 ± 597	1,321 ± 515	1,400 ± 540	913 ± 326	932 ± 490	1,704 ± 579	800 ± 480	1,200 ± 608
Adherence	–	82.4 ± 16.2	–	>80^b^	88.9 ± 20.4	–	79 ± 34	–
Metformin monotherapy, %	85	69	100	100	75	100	88	100

BMI, body mass index.

^a^Creatinine clearance was estimated using the Cockcroft‐Gault formula, except for the PMT1‐EU study in which the MDRD formula was used. ^b^Patients needed to have ≥80% adherence to be included in the study.

As the transport of metformin could depend on its concentration, we next explored possible gene by dose interactions, using a dose as a proxy of metformin concentration. In the meta‐analysis of SNP × dose interaction effects, none of the interactions showed significant effect in the monotherapy group (**Table** 3) or the total group when patients on dual therapy were added to the analysis (**Supplementary Table S3a** online). Likewise, no significant associations were found in the separate meta‐analyses of the effects of variants on metformin response in individuals treated with low (≤1,000 mg) or high daily doses of metformin (>1,000 mg; **Table** 4 and **Supplementary Table S3b** online).

**Table 3:** Meta‐analysis results of single‐nucleotide polymorphism × dose interaction effects in participants on metformin monotherapy

SNP	Effect allele	No. of studies	No. of patients	Continuous dose					Dichotomous dose				
				Beta	SE	*P* value	I^2^	*P*(Q)	Beta	SE	*P* value	I^2^	*P*(Q)
OCT1 R61C	T	5	4,376	0.005	0.019	0.816	0.0	0.965	‐0.005	0.078	0.946	0.0	0.720
OCT1 M420del	del	4	4,297	0.006	0.014	0.669	0.0	0.502	0.043	0.089	0.631	61.8	0.049
OCT1 RF alleles^a^		4	3,986	0.001	0.012	0.923	0.0	0.659	0.041	0.049	0.394	44.0	0.148
OCT1 rs622342	C	5	5,273	0.028	0.010	0.006	39.8	0.156	0.092	0.037	0.014	0.0	0.413
OCT2 A270S	T	6	5,002	0.008	0.017	0.647	30.8	0.205	‐0.003	0.056	0.952	23.0	0.261
MATE1 rs2289669	A	6	4,980	0.014	0.008	0.094	0.2	0.415	0.081	0.036	0.024	0.0	0.485
MATE1 rs2252281	C	4	2,528	0.004	0.015	0.791	0.0	0.505	0.059	0.043	0.177	0.0	0.484
MATE2 rs12943590	A	6	4,273	‐0.008	0.011	0.467	0.0	0.663	0.007	0.044	0.875	0.0	0.827
OCTN1 T306I	T	6	4,647	‐0.005	0.010	0.624	0.0	0.466	‐0.006	0.037	0.879	0.0	0.875
OCTN1 L503F	T	4	4,202	0.025	0.016	0.110	60.4	0.056	0.120	0.085	0.161	76.0	0.006

I^2^, heterogeneity index; MATE, multidrug and toxin extrusion transporter; OCT1, organic cation transporter 1; *P*(Q), *P* value for Cochrane's Q statistic; RF, reduced function; SNP, single‐nucleotide polymorphism.

a Combined genotype for R61C and M420del – number of reduced‐function alleles. A positive beta is a greater glycemic response to metformin associated with the effect allele.

**Table 4:** Meta‐analysis results for the effects of candidate variants in transporter genes on metformin glycemic response in participants on metformin monotherapy, stratified by metformin dose

SNP	Effect allele	No. of studies	Dose ≤1,000 mg						Dose >1,000 mg					
			No. of patients	Beta	SE	*P* value	I^2^	*P*(Q)	No. of patients	Beta	SE	*P* value	I^2^	*P*(Q)
OCT1 R61C	T	5	3,015	0.019	0.038	0.619	0.0	0.855	1,361	0.000	0.000	1.000	0.0	0.719
OCT1 M420del	del	4	2,903	‐0.001	0.050	0.987	55.6	0.080	1,394	0.013	0.040	0.747	44.9	0.142
OCT1 RF alleles^a^		4	2,690	0.006	0.025	0.812	0.2	0.391	1,296	0.032	0.039	0.412	26.1	0.255
OCT1 rs622342	C	5	3,571	‐0.004	0.019	0.837	0.0	0.523	1,702	0.055	0.030	0.064	0.0	0.679
OCT2 A270S	T	6	3,388	0.013	0.029	0.659	0.0	0.749	1,614	0.062	0.082	0.453	50.9	0.070
MATE1 rs2289669	A	6	3,428	‐0.014	0.018	0.452	38.1	0.152	1,552	0.060	0.030	0.046	20.0	0.282
MATE1 rs2252281	C	4	1,629	0.004	0.026	0.877	0.0	0.750	899	‐0.006	0.031	0.843	32.2	0.219
MATE2 rs12943590	A	6	3,009	‐0.012	0.023	0.592	0.0	0.799	1,264	‐0.070	0.088	0.431	93.1	0.000
OCTN1 T306I	T	6	3,116	0.017	0.020	0.401	0.0	0.728	1,531	0.038	0.033	0.251	0.0	0.971
OCTN1 L503F	T	4	2,889	‐0.044	0.020	0.028	35.8	0.197	1,313	‐0.029	0.036	0.434	48.4	0.121

I^2^, heterogeneity index; MATE, multidrug and toxin extrusion transporter; OCT1, organic cation transporter 1; *P*(Q), *P* value for Cochrane's Q statistic; RF, reduced function; SNP, single‐nucleotide polymorphism.

a Combined genotype for R61C and M420del – number of reduced‐function alleles. A positive beta is a greater glycemic response to metformin associated with the effect allele.

We next explored the potential interactions between these transporters that might affect metformin response. The interactions were tested by examining the impact of one SNP in two separate subgroups of participants, homozygotes for the reference, and variant allele of a potential confounding SNP. **Tables** 5 **and** 6 show the results from meta‐analyses of participants on monotherapy for transporters in the liver and kidneys, respectively. **Supplementary Tables S4a and S4b** online show the meta‐analyses results for all participants, including both monotherapy and dual therapy. These exploratory subgroup analyses revealed no significant interactions between the SNPs that affect metformin glycemic response.

**Table 5:** Meta‐analysis results for the effects of individual single‐nucleotide polymorphisms in the subgroups of patients homozygous for the wild‐type or variant allele of possible confounding single‐nucleotide polymorphisms – interactions between metformin liver transporters – monotherapy group

Subgroup	SNP	Effect allele	No. of studies	No. of patients	Beta	SE	*P* value	I^2^	*P*(Q)
0 OCT1 RF alleles^a^	MATE1 rs2289669	A	5	1,736	0.004	0.029	0.879	0.0	0.468
	MATE1 rs2252281	C	4	891	0.028	0.045	0.542	0.0	0.976
2 OCT1 RF alleles	MATE1 rs2289669	A	5	179	‐0.095	0.108	0.380	80.6	0.000
	MATE1 rs2252281	C	4	102	0.167	0.136	0.221	66.5	0.030
OCT1 rs622342 wt/wt	MATE1 rs2289669	A	6	1,899	0.007	0.026	0.789	0.0	0.429
	MATE1 rs2252281	C	4	997	0.046	0.036	0.204	0.0	0.799
OCT1 rs622342 v/v	MATE1 rs2289669	A	6	596	0.084	0.063	0.178	61.7	0.023
	MATE1 rs2252281	C	4	329	0.060	0.051	0.243	35.5	0.200
MATE1 rs2289669 wt/wt	OCT1 RF alleles		5	1,179	0.025	0.036	0.494	31.6	0.211
	OCT1 rs622342	C	6	1,608	0.035	0.049	0.477	53.2	0.058
MATE1 rs2289669 v/v	OCT1 RF alleles		5	292	‐0.008	0.080	0.921	0.0	0.779
	OCT1 rs622342	C	6	527	0.024	0.053	0.653	0.0	0.505
MATE1 rs2252281 wt/wt	OCT1 RF alleles		4	606	0.064	0.052	0.213	0.0	0.837
	OCT1 rs622342	C	4	956	0.040	0.035	0.250	0.0	0.870
MATE1 rs2252281 v/v	OCT1 RF alleles		4	256	‐0.005	0.092	0.957	49.1	0.117
	OCT1 rs622342	C	4	373	0.019	0.054	0.734	0.0	0.575

I^2^, heterogeneity index; MATE, multidrug and toxin extrusion transporter; OCT1, organic cation transporter 1; *P*(Q), *P* value for Cochrane's Q statistic; RF, reduced function; SNP, single‐nucleotide polymorphism; v/v, homozygous variant allele carriers; wt/wt, homozygous wild‐type allele carriers.

a Combined genotype for R61C and M420del ‐ number of RF alleles. A positive beta is a greater glycemic response to metformin associated with the effect allele.

**Table 6:** Meta‐analysis results for the effects of individual single‐nucleotide polymorphisms in the subgroups of patients homozygous for the wild‐type or variant allele of possible confounding single‐nucleotide polymorphisms ‐ interactions between metformin kidney transporters ‐ monotherapy group

Subgroup	SNP	Effect allele	No. of studies	No. of patients	Beta	SE	*P* value	I^2^	*P*(Q)
OCT2 A270S wt/wt	MATE1 rs2289669	A	7	3,869	0.008	0.018	0.673	41.9	0.112
	MATE1 rs2252281	C	5	1,835	‐0.008	0.027	0.756	0.0	0.964
	MATE2 rs12943590	A	7	3,176	‐0.011	0.023	0.642	0.0	0.481
	OCTN1 T306I	T	7	3,642	0.030	0.019	0.118	0.0	0.660
	OCTN1 L503F	T	5	3,437	‐0.040	0.019	0.034	0.0	0.720
OCT2 A270S v/v	MATE1 rs2289669	A	3	59	0.045	0.210	0.831	0.0	0.950
	MATE1 rs2252281	C	2	35	0.575	0.175	0.001	31.8	0.226
	MATE2 rs12943590	A	3	48	0.170	0.337	0.615	0.0	0.557
	OCTN1 T306I	T	3	51	0.164	0.212	0.440	34.6	0.217
	OCTN1 L503F	T	3	50	‐0.017	0.206	0.934	20.3	0.285
MATE1 rs2289669 wt/wt	OCT2 A270S	T	7	1,598	0.021	0.042	0.613	0.0	0.568
	MATE2 rs12943590	A	7	1,288	‐0.066	0.071	0.347	58.9	0.024
	OCTN1 T306I	T	7	1,412	0.044	0.054	0.418	63.4	0.012
	OCTN1 L503F	T	5	1,328	‐0.069	0.029	0.015	0.0	0.884
MATE1 rs2289669 v/v	OCT2 A270S	T	6	495	‐0.066	0.146	0.655	65.0	0.014
	MATE2 rs12943590	A	7	363	0.065	0.052	0.215	0.0	0.529
	OCTN1 T306I	T	7	410	0.138	0.053	0.009	6.4	0.379
	OCTN1 L503F	T	5	365	‐0.156	0.055	0.004	0.0	0.612
MATE1 rs2252281 wt/wt	OCT2 A270S	T	5	862	‐0.014	0.056	0.802	0.0	0.942
	MATE2 rs12943590	A	5	720	0.006	0.046	0.902	9.2	0.354
	OCTN1 T306I	T	5	726	‐0.037	0.042	0.382	47.5	0.107
	OCTN1 L503F	T	4	662	‐0.017	0.046	0.711	19.9	0.290
MATE1 rs2252281 v/v	OCT2 A270S	T	4	329	0.129	0.076	0.087	0.0	0.592
	MATE2 rs12943590	A	5	276	‐0.042	0.061	0.494	0.0	0.829
	OCTN1 T306I	T	5	283	0.013	0.067	0.853	22.1	0.274
	OCTN1 L503F	T	4	262	‐0.011	0.074	0.884	0.0	0.650
MATE2 rs12943590 wt/wt	OCT2 A270S	T	7	1,964	0.001	0.036	0.978	22.4	0.259
	MATE1 rs2289669	A	7	1,980	‐0.010	0.023	0.673	0.0	0.454
	MATE1 rs2252281	C	5	1,014	0.051	0.034	0.138	0.0	0.661
	OCTN1 T306I	T	7	1,828	0.043	0.026	0.095	39.7	0.127
	OCTN1 L503F	T	5	1,636	‐0.050	0.027	0.062	26.4	0.246
MATE2 rs12943590 v/v	OCT2 A270S	T	7	298	‐0.017	0.088	0.849	0.0	0.830
	MATE1 rs2289669	A	7	300	0.183	0.115	0.112	51.3	0.055
	MATE1 rs2252281	C	5	139	0.089	0.097	0.357	0.0	0.534
	OCTN1 T306I	T	7	279	‐0.071	0.056	0.203	0.0	0.744
	OCTN1 L503F	T	5	241	0.080	0.058	0.168	0.0	0.455
OCTN1 T306I wt/wt	OCT2 A270S	T	7	1,806	0.081	0.041	0.048	0.0	0.557
	MATE1 rs2289669	A	7	1,728	‐0.020	0.028	0.469	0.0	0.554
	MATE1 rs2252281	C	5	827	‐0.001	0.040	0.976	35.2	0.187
	MATE2 rs12943590	A	7	1,426	0.018	0.033	0.581	0.0	0.630
OCTN1 T306I v/v	OCT2 A270S	T	6	634	‐0.017	0.067	0.803	31.7	0.198
	MATE1 rs2289669	A	7	609	‐0.023	0.040	0.571	25.7	0.232
	MATE1 rs2252281	C	5	267	0.049	0.122	0.690	63.7	0.027
	MATE2 rs12943590	A	7	541	‐0.145	0.094	0.121	63.1	0.012
OCTN1 L503F wt/wt	OCT2 A270S	T	5	1,367	‐0.040	0.047	0.394	41.8	0.143
	MATE1 rs2289669	A	5	1,274	‐0.019	0.031	0.548	0.0	0.724
	MATE1 rs2252281	C	4	583	‐0.059	0.063	0.344	42.2	0.158
	MATE2 rs12943590	A	5	1,114	‐0.049	0.057	0.394	53.5	0.072
OCTN1 L503F v/v	OCT2 A270S	T	5	816	0.106	0.063	0.095	40.2	0.153
	MATE1 rs2289669	A	5	768	‐0.042	0.045	0.354	0.0	0.982
	MATE1 rs2252281	C	4	337	0.017	0.065	0.793	5.6	0.365
	MATE2 rs12943590	A	5	584	0.031	0.053	0.561	0.0	0.857

A positive beta is a greater glycemic response to metformin associated with the effect allele.

I^2^, heterogeneity index; MATE, multidrug and toxin extrusion transporter; OCT1, organic cation transporter 1; *P*(Q), *P* value for Cochrane's Q statistic; SNP, single‐nucleotide polymorphism; v/v, homozygous variant allele carriers; wt/wt, homozygous wild‐type allele carriers.

In the supplemental locus‐wise meta‐analysis of the association of all common SNPs in transporter gene regions with metformin glycemic response, none of the variants showed significant signal after correction for multiple testing (*P* > 1.6 × 10^−4^; **Supplementary Figure S1**).

## DISCUSSION

Despite the established role of cation‐selective transporters in metformin pharmacokinetics, polymorphisms in these transporters showed no significant impact on glycemic response to metformin in this large study of patients with T2D. This meta‐analysis had 80% power to detect an allelic effect of HbA_1c_ reduction >0.14% (1.5 mmol/mol) for any of the candidate SNPs at the nominal significance level of α = 0.005. Thus, none of the SNPs reported as being associated with metformin response in previous literature is likely to have an allelic impact on HbA_1c_ reduction of >0.14%. Furthermore, it is unlikely that other SNPs, such as *cis*‐regulatory variants, in these genes could have a significant impact on metformin glycemic response, as shown by a locus‐wise meta‐analysis of all common SNPs within genes' proximity in 6,964 patients.

Our findings contrast to most of those previously reported in healthy subjects,^15^, ^16^, ^17^, ^18^ although it is in keeping with a recent study showing no impact of the OCT1 genotype on the glucose production in fasting healthy subjects.^32^ This may reflect the fact that our study was conducted on patients with T2D and, as such, we were able to assess HbA_1c_ reduction rather than other surrogates of metformin response. Some^18^, ^20^, ^21^, ^23^, ^24^, ^25^ but not all^19^ previous studies have reported an association of a variant altering metformin transport and glycemic response to metformin in patients with T2D. These studies have varied in size but have generally been small, and have varied in their definition of glycemic response to metformin and analytical approaches. In this MetGen meta‐analysis, we included all studies that have previously explored associations between transporter variants and glycemic response to metformin in patients with T2D of European ancestry with both positive and negative findings,^18^, ^19^, ^20^, ^21^, ^22^, ^23^, ^24^, ^25^ and also other cohorts for which results have not been published. In this way, we have reduced the chance of reporting bias and maximized the power. In addition, we have reduced heterogeneity by aligning metformin response definitions, models, and covariates for all studies included in the meta‐analysis. Our results suggest that metformin transporters do not have a significant role in how patients with T2D respond to metformin therapy.

Transporters exhibit asymmetry in their kinetic properties; thus, for facilitated transporters that are bidirectional, the direction of the transport will depend on the substrate concentration.^33^, ^34^ Systemic plasma levels of metformin are dependent on dose; therefore, in our secondary analyses, we analyzed dose × SNP interactions and assessed the effect of the studied variants separately in individuals prescribed low (≤1,000 mg) or high (>1,000 mg) doses of the drug. We did not find significant impact of the analyzed interactions on metformin glycemic response, and, accordingly, significant association between any variant and response in the dose‐stratified analysis. However, we used the prescribed dose as a proxy of metformin concentration. There were differences in the characteristics of patients between the cohorts, and, for instance, older age and lower estimated glomerular filtration rate in the Rotterdam cohort could result in higher metformin serum levels, despite being prescribed lower doses. In addition, data on drug adherence were available only in four studies, although all studies were adjusted for creatinine clearance and other known clinical covariates, which could influence metformin response.

Studies of small cohorts have previously reported gene‐gene interactions affecting glycemic response to metformin.^18^, ^22^ Here, we explored whether such interactions could explain the lack of association between metformin response and transporter variants. The exploratory analyses of SNP × SNP interactions, assessed as the impact of studied polymorphisms on metformin response in the subgroups of patients homozygous for possibly confounding SNPs, did not show any significant effects. However, these subgroup analyses had substantially less power to detect moderate effects due to the smaller sample sizes, especially for the rarer SNPs. Larger studies would be needed to detect these effects and to explore possible but more complex multiple gene‐gene interactions.

This is the largest metformin pharmacogenetic study reported to date, despite a few limitations due to the need to align cohorts. Our finding that there is no significant role for metformin transporter variants in mediating glycemic response to metformin challenges our understanding of metformin action in patients with T2D chronically treated with metformin. For example, there is increasing recognition that metformin works presystemically in the gut, via a number of mechanisms, to improve glycemia.^6^, ^35^, ^36^, ^37^ Indeed, a recent delayed release metformin achieves low systemic metformin concentrations but is effective at lowering blood glucose in patients with T2D.^38^ It is also possible that the influence of the analyzed transporter gene variants is less prominent clinically than expected due to the redundancy of transporters *in vivo*.^27^ If one or more transporters have reduced function, other transporters may take over their roles and mediate metformin uptake or efflux from the organs. In addition, there might be more membrane transporters of metformin yet to be identified that could have a role in its absorption, distribution, and elimination. In the current study, we focused on the candidate variants in the membrane transporter genes that have been associated previously with metformin pharmacokinetics or response. However, in addition to liver and kidney transporters, transporters in the intestine may play a significant role in metformin levels. Recent studies have suggested that other transporters, which were not the subject of this study, play an important role in metformin absorption,^34^, ^39^, ^40^, ^41^ and there still may be unidentified cation transporters in the intestine involved in metformin absorption, which could also affect metformin response. It should also be noted that variants in transporters in various tissues may play opposing roles. For example, OCT1 could mediate basolateral flux of metformin from enterocytes to the portal circulation^7^, ^42^ and across the sinusoidal membrane of hepatocytes.^7^ Thus, a reduced function OCT1 genetic variant may result in increased concentrations in enterocytes and decreased concentrations in hepatocytes.

Although the current study demonstrates that genetic variants in transporters that play a role in metformin pharmacokinetics have no significant effect on metformin glycemic response in large cohorts of patients with diabetes, there are some limitations to our study. First, despite the large sample size used in the current study, we did not have the statistical power to detect an allelic effect size for HbA_1c_ reduction smaller than 0.06–0.14% (0.7–1.5 mmol/mol, depending on SNP frequency), however, these small effect sizes may be unlikely to have any clinical importance. Second, the meta‐analysis included population‐based observational studies, which could be confounded by a number of factors, such as patient adherence or frequency of HbA_1c_ measurements in the cohort. However, in four cohorts, including two largest cohorts, GoDARTS and PMT2‐EU, adherence could be calculated from the drug dispensing records, and was added as a covariate in the model. Likewise, although we could not completely harmonize the definition of on‐treatment HbA_1c_ due to different frequency of HbA_1c_ measurements available in the cohorts, four studies, including two of the largest studies, used minimal HbA_1c_ achieved within 18 months as outcome. Third, concomitant medications, which affect metformin transport, are well established to cause changes in its pharmacokinetics,^43^ and may obscure effects of genetic variants on metformin response. Information on prescription and over‐the‐counter drugs that affect metformin pharmacokinetics, such as cimetidine, were not gathered in the current study. In addition, several previous studies have shown effects of polymorphisms in transporters on metformin response in multi‐ethnic cohorts, including many individuals from non‐European ancestries.^18^, ^24^ Genetic variants may have different effect sizes on drug response in individuals from different ethnic backgrounds. Further studies are needed to test the effects of these variants on metformin response in individuals from non‐European backgrounds. Finally, recent studies suggest that genetic variants in transcription factors that affect expression of several metformin transporters may have larger effect sizes on metformin response than genetic variants in the individual transporters themselves,^44^ underscoring the need to understand not only the mechanisms of metformin transport in various tissues, but the proteins that modulate their activity and expression.

In conclusion, in our large meta‐analysis, including almost 8,000 individuals across 10 international cohorts of the MetGen Consortium, variants in metformin transporter genes have shown no relevant contribution to variability in metformin response in patients with T2D, although we cannot rule out gene‐concentration or more complex multiple gene‐gene interactions that may be required to account for transporter redundancy. As has been recognized now for a number of years in disease and complex trait genetics, this study shows the importance of large sample sizes, usually only available to international consortia, for robust pharmacogenetic studies. Future even larger consortia efforts are required to corroborate these findings and to unravel genetic variations that could be used as better predictors for personalized metformin therapy.

## METHODS

### Studies

The MetGen Consortium consists of research groups from Europe and the United States with available data for studies of metformin pharmacogenetics from population observational studies and clinical trials. The 10 MetGen cohorts with available data for the study of metformin transporter gene variants are presented in **Supplementary Table S5** online. The studies were approved by relevant institutional review boards, and all participants gave written informed consent.

### Single‐nucleotide polymorphism selection and genotyping

Nine SNPs in five transporter genes, reported to be associated with metformin response or pharmacokinetics in previous studies, were included in the meta‐analysis (**Table** 1). The numbers of available SNPs, genotyping methods, and the minor allele frequencies in each cohort are provided in **Supplementary Table S6** online. All SNPs were in line with Hardy‐Weinberg equilibrium (*P* > 0.01). The variants were first analyzed individually for association with metformin response. The reduced‐function OCT1 polymorphisms, R61C and M420del, were analyzed individually, and also together as a combined genotype, according to the number of haplotypes carrying reduced‐function alleles: 0, 1, or 2, in line with previous studies in patients of European ancestry.^23^, ^45^, ^46^

### Assessment of metformin response

Metformin response was defined as a reduction in HbA_1c_ during treatment with metformin: pretreatment minus on‐treatment HbA_1c_. As such, a positive β in the regression models indicates an association of the effect allele with greater glycemic response to metformin. Inclusion criteria for all participants included in the meta‐analysis were continuous treatment with metformin for at least 3 months, pretreatment HbA_1c_ measured within 6 months prior to metformin therapy and <14%, measurement of on‐treatment HbA_1c_ within 18 months of metformin commencement, and no treatment with other glucose‐lowering agents except stable sulfonylurea treatment before and during metformin therapy. Hence, two cohorts from randomized controlled trials, HOME and SDDS studies, in which metformin was added to insulin therapy, were not included in the meta‐analysis, and the results are shown separately.

On‐treatment HbA_1c_ was defined as minimal HbA_1c_ measured within 18 months of metformin commencement in the GoDARTS, PMT2‐EU, Riga, and Rotterdam studies, as HbA_1c_ measured after 6 months (Košice and Sarajevo studies) or 12 months (DCS study) of metformin treatment, and as HbA_1c_ measured within the first 3–9 months of metformin therapy in the PMT1‐EU study.

### Statistical analysis

In each cohort, the effects of individual SNPs on metformin response were assessed in additive genetic model using linear regression with reduction of HbA_1c_ as outcome (primary analysis). The pretreatment HbA_1c_, metformin daily dose, adherence, creatinine clearance, baseline gap (time between pretreatment HbA_1c_ measurement and start of metformin therapy), and treatment group (metformin prescribed as monotherapy or dual therapy – metformin added to stable sulfonylurea treatment), were added as covariates, when available/applicable (**Supplementary Table S7** online). In the HOME study, analyses were adjusted for pretreatment HbA_1c_, metformin daily dose and creatinine clearance, and in the SDDS study, pretreatment HbA_1c_ and randomization group^23^ were added as covariates.

As the effects of transporter SNPs could depend on metformin level, or they could be confounded by the effect of other variants, secondary analyses were performed to explore possible gene‐dose and gene‐gene interactions. For gene‐dose interactions, we first examined interaction models that included SNP × dose interaction term with dose as a continuous variable and then dose coded as a dichotomized variable (low or high dose) in the following model: HbA_1c_ reduction ∼ pre‐treatment HbA_1c_ + adherence + creatinine clearance + baseline gap + treatment group + dose + SNP + SNP × dose.

Next, we assessed the association of SNPs with metformin response separately for patients taking low (≤1,000 mg) or high doses (>1,000 mg) of metformin. The cutoff value of 1,000 mg was chosen based on the median dose in the largest cohort. The analyses were performed using the same basic regression model: HbA_1c_ reduction ∼ pre‐treatment HbA_1c_ + adherence + creatinine clearance + baseline gap + treatment group + dose + SNP.

To examine potential interactions between the variants, additional exploratory analyses of the effects of individual SNPs were carried out in the subgroups of individuals who were homozygous for wild‐type allele and of the individuals who were homozygous for variant allele of possibly confounding SNPs, assuming that impact of the variants could be more pronounced in more extreme genotype groups. Possible 1 × 1 interactions between SNPs in metformin liver transporters (OCT1 and MATE1), and SNPs in metformin kidney transporters (OCT2, MATE1, MATE2‐K, and OCTN1) were explored.

All analyses were carried out for the total group of patients treated with metformin, including monotherapy and dual therapy group (metformin added to stable sulfonylurea therapy), and also separately for the metformin monotherapy group. The results from individual studies were combined in the meta‐analysis using PLINK software under fixed‐effects model (http://pngu.mgh.harvard.edu/purcell/plink/).^47^ For gene‐dose interaction models, estimates of SNP × dose interaction terms were pooled in the meta‐analysis.^48^ Heterogeneity across studies was assessed using the Cochran Q test and the I^2^ heterogeneity index. In case the substantial heterogeneity was detected (*P* value for Cochran Q test <0.10), the results of the random‐effects model were presented. Power analyses were performed using Quanto (http://biostats.usc.edu/Quanto.html). Forest plots were created using RevMan 5.3 software (Review Manager, The Nordic Cochrane Centre, The Cochrane Collaboration).

As the primary analysis included nine SNPs and one combined OCT1 genotype, a nominal statistical significance threshold was set to 5 × 10^−3^ (*P* = 0.05/10), and a study‐wise statistical significance threshold to 2 × 10^−4^ to correct for the overall number of tests performed (*P* = 0.05/236).

In addition to our main hypothesis, we performed a supplemental, locus‐wise meta‐analysis of all common SNPs within the proximity of the transporter genes to examine the possibility that original associations reported in previous studies might be driven by other SNPs in these gene regions. A total of 3,471 variants with minor allele frequency ≥1% were tested for association with metformin glycemic response in 6,964 participants from two MetGen cohorts with available genome‐wide association study imputed data, the GoDARTS and PMT2‐EU. The details of genotyping, quality control, imputation, association analysis, and meta‐analysis were described in our previous study.^49^ The experiment‐wise significance threshold for this supplemental analysis was derived from 10,000 permutations of the phenotype in the GoDARTS data,^47^ which gave a 95% cutoff for significance of *P* = 1.6 × 10^−4^.

## AUTHOR CONTRIBUTIONS

T.D., K.Z., K.M.G., and E.R.P. wrote the manuscript. T.D., K.M.G., and E.R.P. designed the research. T.D., K.Z., S.W.Y., N.v.L., C.E.d.K., M.J., S.G., L.Z., M.M.H.C., M.O., R.T., M.K., M.M.H., A.A.v.d.H., L.K., V.P., A.K., K.B., J.K., S.S., I.T., B.H.S., C.N.A.P., L.M.t.H., K.M.G., and E.R.P. performed the research. T.D., K.Z., S.W.Y., N.v.L., C.E.d.K., M.J., S.G., L.Z., M.M.H.C., and M.O. analyzed the data.

## CONFLICT OF INTEREST

The authors declared no conflict of interest.

## Supporting information

Supporting Information
