# Supplementary material for: Variants in Pharmacokinetic Transporters and Glycemic Response to Metformin: A Metgen Meta‐Analysis
Source: Clin Pharmacol Ther. 2017 Feb 3;101(6):763–72. doi: 10.1002/cpt.567 (PMC5425333; doi:10.1002/cpt.567)
Supplement: Supplementary file 1 — Supporting Information [file CPT-101-763-s001.pdf]

Supplementary Table 1. MetGen cohorts.

Cohort	Ethnicity	Country	Reference	Study Design
DCS	European	Netherlands	van Leeuwen et al., 2012[1]	Population, observational, EMR-linked
GoDARTS	European	Scotland, UK	Zhou et al., 2011[2]	Population, observational, EMR-linked
HOME	European	Netherlands	Kooy et al., 2009[3]	RCT, metformin added to insulin
Košice	European	Slovakia	Tkáč et al., 2013[4]	Population, observational, retrospective
PMT1-EU	European American	USA	Choi et al., 2011[5]; Stocker et al., 2013[6]; Goswami et al., 2014[7]	Population, observational, EMR-linked
PMT2-EU*	European American	USA	Hoffmann et al., 2011[8]; Banda et al., 2015[9]; Kvale et al., 2015[10]	Population, observational, EMR-linked
Riga	European	Latvia	Tarasova et al., 2012[11]	Population, observational, retrospective
Rotterdam	European	Netherlands	Becker et al., 2009[12]	Population, observational, EMR-linked
Sarajevo	European	Bosnia and Herzegovina	Dujic et al., 2016[13]	Population, observational, prospective
SDDS	European	Denmark	Christensen et al., 2011[14]	RCT, metformin added to insulin and rosiglitazone/placebo

DCS, The Hoorn Diabetes Care System cohort study. GoDARTS, Genetics of Diabetes Audit and Research Tayside Study. HOME, Hyperinsulinemia: the Outcome of its Metabolic Effects trial. PMT, Pharmacogenomics of Membrane Transporters studies. SDDS, South Danish Diabetes Study. EMR, electronic medical record. RCT, randomized controlled trial. *Participants from the PMT2-EU cohort were selected from the Genetic Epidemiology Research on Adult Health and Aging (GERA) cohort, a subsample of the Kaiser Permanente Research Program on Genes, Environment, and Health (RPGEH).

Supplementary Table 2. Genotyping platforms and minor allele frequencies of analysed SNPs in the individual studies.

		DCS	GoDARTS	HOME	Košice	PMT1-EU	PMT2-EU	Riga	Rotterdam	Sarajevo	SDDS
Genotyping Platform		Array + TaqMan	Array + TaqMan		TaqMan	Array + TaqMan	Array	Array	Array	TaqMan	TaqMan + Sequencing
Number of available SNPs		9	9	3	5	9	8 *	3	8 *	7	6
Minor allele frequencies	Minor allele										
OCT1 R61C	T	0.08	0.06	0.08	-	0.06	0.08	0.07	0.07	0.11	0.07
OCT1 M420del	del	0.18	0.19	-	-	0.15	0.18	-	-	0.16	0.18
OCT1 rs622342	C	0.36	0.39	0.40	0.25	0.33	0.37	-	0.33	-	0.39
OCT2 A270S	T	0.13	0.11	-	0.06	0.12	0.10	0.08	0.11	0.04	0.10
MATE1 rs2289669	A	0.43	0.38	0.37	0.45	0.34	0.39	0.37	0.36	0.39	0.43
MATE1 rs2252281	C	0.38	0.37	-	-	0.41	-	-	0.37	0.40	0.41
MATE2-K rs12943590	A	0.27	0.27	-	0.33	0.27	0.30	-	0.26	0.39	-
OCTN1 T306I	T	0.33	0.36	-	0.44	0.36	0.39	-	0.32	0.41	-
OCTN1 L503F	T	0.44	0.44	-	-	0.30	0.43	-	0.37	-	-

* SNPs were imputed using SNPTEST (PMT2-EU) or MACH software (Rotterdam) and the 1,000 Genomes reference panel.

Supplementary Table 3. Clinical factors added as covariates in individual studies.

Covariate	DCS	GoDARTS	Košice	PMT1-EU	PMT2-EU	Riga	Rotterdam	Sarajevo
Pre-treatment HbA_{1c}	Y	Y	Y	Y	Y	Y	Y	Y
Daily dose	Y	Y	Y	Y	Y	Y	Y	Y
Adherence	N	Y	N	N	Y	N	Y	N
Creatinine clearance	Y	Y	Y	Y	Y	Y	Y	Y
Baseline gap[a]	NA	Y	NA	Y	Y	Y	Y	NA
Treatment group[b]	Y	Y	NA	NA	Y	NA	Y	NA

Y, yes. N, not available. NA, not applicable. [a] Time between pre-treatment HbA_{1c} measurement and start of metformin therapy. [b] Metformin prescribed as monotherapy or dual therapy (metformin added to stable sulfonylurea treatment).

Supplementary Table 4. Meta-analysis results for the effects of candidate variants in transporters genes on metformin glycaemic response in the total group of patients (monotherapy and dual therapy).

SNP	Effect allele	n	N	Beta	SE	*p*	I^2	*p*(Q)
OCT1 R61C	T	7	6065	0.009	0.028	0.740	0.0	0.939
OCT1 M420del	del	5	5973	0.013	0.020	0.506	0.0	0.980
OCT1 RF alleles*		5	5559	0.015	0.018	0.402	0.0	0.950
OCT1 rs622342	C	6	7081	0.008	0.014	0.573	0.0	0.502
OCT2 A270S	T	8	6813	0.026	0.022	0.241	27.0	0.213
MATE1 rs2289669	A	8	6736	0.0004	0.013	0.976	4.6	0.395
MATE1 rs2252281	C	5	3346	0.016	0.020	0.426	0.0	0.499
MATE2 rs12943590	A	7	5530	-0.009	0.017	0.605	0.0	0.569
OCTN1 T306I	T	7	6362	-0.001	0.014	0.971	0.0	0.512
OCTN1 L503F	T	5	5818	-0.003	0.015	0.843	0.0	0.645

n, number of studies included in the meta-analysis; N, overall number of patients; I^2, heterogeneity index; p(Q), p-value for Cochrane's Q statistic. *Combined genotype for R61C and M420del - number of reduced-function (RF) alleles. A positive beta is a greater glycaemic response to metformin associated with the effect allele.

Supplementary Table 5. Association of individual SNPs with metformin glycaemic response in the HOME and SDDS studies.

SNP	Effect allele	HOME				SDDS			
		N	Beta	SE	*p*	N	Beta	SE	*p*
OCT1 R61C	T	163	0.063	0.201	0.753	149	-0.010	0.186	0.960
OCT1 M420del	del	-	-	-	-	148	0.060	0.133	0.650
OCT1 RF alleles*		-	-	-	-	148	0.040	0.120	0.720
OCT1 rs622342	C	163	-0.019	0.113	0.863	148	-0.180	0.102	0.072
OCT2 A270S	T	-	-	-	-	149	-0.320	0.161	0.050
MATE1 rs2289669	A	163	0.099	0.118	0.402	149	-0.030	0.094	0.760
MATE1 rs2252281	C	-	-	-	-	146	0.070	0.092	0.440

N, number of patients. *Combined genotype for R61C and M420del - number of reduced-function (RF) alleles. A positive beta is a greater glycaemic response to metformin associated with the effect allele.

Supplementary Table 6a. Meta-analysis results of SNP x dose interaction effects in all participants treated with metformin (monotherapy and dual therapy).

SNP	Effect allele	n	N	Continuous dose					Dichotomous dose				
				Beta	SE	*p*	I^2	p(Q)	Beta	SE	*p*	I^2	p(Q)
OCT1 R61C	T	5	5884	-0.004	0.017	0.797	0.0	0.929	-0.032	0.065	0.623	0.0	0.681
OCT1 M420del	del	4	5847	0.009	0.012	0.430	0.0	0.855	0.066	0.042	0.115	15.1	0.317
OCT1 RF alleles*		4	5433	0.003	0.010	0.794	0.0	0.951	0.043	0.040	0.287	0.0	0.848
OCT1 rs622342	C	5	6915	0.023	0.009	0.009	42.6	0.138	0.091	0.032	0.004	0.0	0.496
OCT2 A270S	T	6	6586	0.010	0.015	0.481	38.0	0.153	0.017	0.048	0.734	15.9	0.312
MATE1 rs2289669	A	6	6506	0.011	0.008	0.136	0.0	0.550	0.056	0.031	0.073	0.0	0.417
MATE1 rs2252281	C	4	3223	0.004	0.012	0.723	0.0	0.884	0.037	0.039	0.344	0.0	0.860
MATE2 rs12943590	A	6	5405	-0.006	0.010	0.579	0.0	0.648	0.011	0.039	0.768	0.0	0.842
OCTN1 T306I	T	6	6171	0.001	0.009	0.927	12.1	0.338	0.008	0.032	0.801	0.0	0.875
OCTN1 L503F	T	4	5655	0.013	0.008	0.114	40.3	0.170	0.066	0.053	0.218	54.2	0.088

n, number of studies included in the meta-analysis; N, overall number of patients; I^2, heterogeneity index; p(Q), p-value for Cochrane's Q statistic.
*Combined genotype for R61C and M420del - number of reduced-function (RF) alleles. A positive beta is a greater glycaemic response to metformin associated with the effect allele.

Supplementary Table 6b. Meta-analysis results for the effects of candidate variants in transporters genes on metformin glycaemic response in all participants treated with metformin (monotherapy and dual therapy) - stratified by metformin dose.

SNP	Effect allele	n	Dose <=1000 mg						Dose > 1000 mg					
			N	Beta	SE	*p*	I^2	p(Q)	N	Beta	SE	*p*	I^2	p(Q)
OCT1 R61C	T	5	3898	0.017	0.034	0.616	0.0	0.931	1986	-0.019	0.054	0.728	0.0	0.757
OCT1 M420del	del	4	3818	-0.001	0.024	0.967	44.0	0.148	2029	0.046	0.034	0.175	0.0	0.660
OCT1 RF alleles*		4	3525	0.004	0.022	0.860	0.0	0.452	1908	0.040	0.032	0.210	0.0	0.999
OCT1 rs622342	C	5	4561	-0.013	0.017	0.436	25.1	0.254	2354	0.045	0.026	0.085	0.0	0.826
OCT2 A270S	T	6	4354	0.015	0.026	0.575	0.0	0.524	2232	0.076	0.075	0.312	53.3	0.057
MATE1 rs2289669	A	6	4374	-0.003	0.016	0.854	25.4	0.243	2132	0.037	0.026	0.160	35.8	0.169
MATE1 rs2252281	C	4	2026	0.019	0.024	0.440	0.0	0.712	1197	0.028	0.036	0.441	0.0	0.601
MATE2 rs12943590	A	6	3719	-0.009	0.020	0.643	0.0	0.793	1686	-0.023	0.029	0.437	0.0	0.843
OCTN1 T306I	T	6	4010	0.001	0.018	0.974	0.0	0.589	2161	0.015	0.027	0.592	0.0	0.683
OCTN1 L503F	T	4	3753	-0.018	0.018	0.307	0.0	0.463	1902	0.026	0.027	0.336	23.6	0.270

n, number of studies included in the meta-analysis; N, overall number of patients; I^2, heterogeneity index; p(Q), p-value for Cochrane's Q statistic.
*Combined genotype for R61C and M420del - number of reduced-function (RF) alleles. A positive beta is a greater glycaemic response to metformin associated with the effect allele.

Supplementary Table 7a. Meta-analysis results for the effects of individual SNPs in the subgroups of patients homozygous for the wild-type (wt/wt) or variant allele (v/v) of possible confounding SNPs - interactions between metformin liver transporters - all patients (monotherapy and dual therapy).

Subgroup	SNP	Effect allele	n	N	Beta	SE	*p*	I^2	p(Q)
0 OCT1 RF alleles*	MATE1 rs2289669	A	5	2332	0.0002	0.026	0.994	0.0	0.734
	MATE1 rs2252281	C	4	1199	0.034	0.038	0.367	0.0	0.972
2 OCT1 RF alleles	MATE1 rs2289669	A	5	246	-0.112	0.108	0.300	83.4	0.000
	MATE1 rs2252281	C	4	139	0.176	0.129	0.173	67.5	0.026
OCT1 rs622342 wt/wt	MATE1 rs2289669	A	6	2450	0.006	0.023	0.812	14.3	0.323
	MATE1 rs2252281	C	4	1256	0.039	0.032	0.226	0.0	0.762
OCT1 rs622342 v/v	MATE1 rs2289669	A	6	832	0.064	0.063	0.305	67.9	0.008
	MATE1 rs2252281	C	4	431	0.046	0.049	0.351	32.3	0.219
MATE1 rs2289669 wt/wt	OCT1 RF alleles		5	1600	0.039	0.032	0.220	14.4	0.322
	OCT1 rs622342	C	6	2126	0.002	0.040	0.961	47.7	0.089
MATE1 rs2289669 v/v	OCT1 RF alleles		5	387	0.035	0.067	0.596	0.0	0.733
	OCT1 rs622342	C	6	661	-0.024	0.045	0.594	0.0	0.859
MATE1 rs2252281 wt/wt	OCT1 RF alleles		4	829	0.030	0.044	0.506	0.0	0.900
	OCT1 rs622342	C	4	1228	0.018	0.031	0.570	0.0	0.856
MATE1 rs2252281 v/v	OCT1 combined		4	333	-0.091	0.080	0.257	49.6	0.114
	OCT1 RF alleles	C	4	466	-0.006	0.049	0.897	0.0	0.486

n, number of studies included in the meta-analysis; N, overall number of patients; I^2, heterogeneity index; p(Q), p-value for Cochrane's Q statistic.
*Combined genotype for R61C and M420del - number of reduced-function (RF) alleles. A positive beta is a greater glycaemic response to metformin associated with the effect allele.

Supplementary Table 7b. Meta-analysis results for the effects of individual SNPs in the subgroups of patients homozygous for the wild-type (wt/wt) or variant allele (v/v) of possible confounding SNPs - interactions between metformin kidney transporters - all patients (monotherapy and dual therapy).

Subgroup	SNP	Effect allele	n	N	Beta	SE	*p*	I^2	p(Q)
OCT2 A270S wt/wt	MATE1 rs2289669	A	7	5070	0.008	0.016	0.644	36.5	0.150
	MATE1 rs2252281	C	5	2337	0.002	0.024	0.924	0.0	0.830
	MATE2 rs12943590	A	7	4016	-0.005	0.020	0.812	0.0	0.525
	OCTN1 T306I	T	7	4813	0.011	0.017	0.537	0.0	0.527
	OCTN1 L503F	T	5	4601	-0.006	0.016	0.700	7.9	0.362
OCT2 A270S v/v	MATE1 rs2289669	A	3	77	-0.073	0.146	0.619	0.0	0.876
	MATE1 rs2252281	C	2	45	0.361	0.158	0.022	0.0	0.923
	MATE2 rs12943590	A	3	60	0.056	0.239	0.816	0.0	0.484
	OCTN1 T306I	T	3	66	0.069	0.182	0.706	40.8	0.185
	OCTN1 L503F	T	3	64	0.119	0.166	0.475	38.1	0.199
MATE1 rs2289669 wt/wt	OCT2 A270S	T	7	2117	0.025	0.037	0.489	8.2	0.366
	MATE2 rs12943590	A	7	1617	-0.053	0.056	0.350	49.0	0.068
	OCTN1 T306I	T	7	1891	0.049	0.050	0.324	62.9	0.013
	OCTN1 L503F	T	5	1804	-0.044	0.026	0.085	0.0	0.874
MATE1 rs2289669 v/v	OCT2 A270S	T	6	629	-0.018	0.127	0.888	62.8	0.020
	MATE2 rs12943590	A	7	419	0.073	0.049	0.139	9.0	0.360
	OCTN1 T306I	T	7	524	0.082	0.048	0.087	6.5	0.378
	OCTN1 L503F	T	5	477	-0.107	0.047	0.024	0.0	0.891
MATE1 rs2252281 wt/wt	OCT2 A270S	T	5	1111	0.032	0.050	0.514	0.0	0.883
	MATE2 rs12943590	A	5	869	-0.010	0.043	0.814	0.0	0.520
	OCTN1 T306I	T	5	960	-0.021	0.038	0.581	47.2	0.108
	OCTN1 L503F	T	4	880	-0.004	0.040	0.928	27.4	0.247
MATE1 rs2252281 v/v	OCT2 A270S	T	4	414	0.123	0.069	0.074	0.0	0.528
	MATE2 rs12943590	A	5	325	-0.031	0.057	0.581	0.0	0.757
	OCTN1 T306I	T	5	369	-0.022	0.057	0.695	37.2	0.173

	OCTN1 L503F	T	4	340	-0.009	0.064	0.885	0.0	0.824
MATE2 rs12943590 wt/wt	OCT2 A270S	T	7	2487	0.007	0.034	0.834	41.9	0.112
	MATE1 rs2289669	A	7	2479	0.003	0.020	0.903	0.0	0.508
	MATE1 rs2252281	C	5	1210	0.058	0.033	0.075	0.0	0.591
	OCTN1 T306I	T	7	2344	0.039	0.023	0.093	40.8	0.119
	OCTN1 L503F	T	5	2127	-0.024	0.023	0.301	37.3	0.173
MATE2 rs12943590 v/v	OCT2 A270S	T	7	392	-0.065	0.074	0.379	0.0	0.499
	MATE1 rs2289669	A	7	388	0.183	0.099	0.064	63.1	0.013
	MATE1 rs2252281	C	5	173	0.087	0.086	0.314	10.9	0.344
	OCTN1 T306I	T	7	371	-0.045	0.050	0.366	0.0	0.465
	OCTN1 L503F	T	5	327	0.058	0.050	0.251	18.7	0.296
OCTN1 T306I wt/wt	OCT2 A270S	T	7	2373	0.056	0.037	0.126	1.7	0.412
	MATE1 rs2289669	A	7	2260	-0.006	0.028	0.819	3.4	0.400
	MATE1 rs2252281	C	5	1077	0.028	0.036	0.447	15.5	0.316
	MATE2 rs12943590	A	7	1826	0.028	0.029	0.337	0.0	0.690
OCTN1 T306I v/v	OCT2 A270S	T	6	824	-0.042	0.113	0.713	48.9	0.081
	MATE1 rs2289669	A	7	788	-0.014	0.023	0.537	34.9	0.162
	MATE1 rs2252281	C	5	338	0.018	0.120	0.879	66.6	0.018
	MATE2 rs12943590	A	7	678	-0.131	0.090	0.147	65.3	0.008
OCTN1 L503F wt/wt	OCT2 A270S	T	5	1832	-0.012	0.076	0.877	57.9	0.050
	MATE1 rs2289669	A	5	1710	-0.024	0.028	0.378	0.0	0.732
	MATE1 rs2252281	C	4	770	-0.044	0.053	0.412	44.8	0.143
	MATE2 rs12943590	A	5	1437	-0.056	0.057	0.324	57.1	0.054
OCTN1 L503F v/v	OCT2 A270S	T	5	1080	0.072	0.056	0.202	47.3	0.108
	MATE1 rs2289669	A	5	1012	-0.012	0.040	0.763	0.0	0.991
	MATE1 rs2252281	C	4	432	0.030	0.058	0.603	22.4	0.276
	MATE2 rs12943590	A	5	767	0.037	0.045	0.419	0.0	0.759

n, number of studies included in the meta-analysis; N, overall number of patients; I^2, heterogeneity index; p(Q), p-value for Cochrane's Q statistic. A positive beta is a greater glycaemic response to metformin associated with the effect allele.

References

1. van Leeuwen, N. *et al.* A gene variant near ATM is significantly associated with metformin treatment response in type 2 diabetes: a replication and meta-analysis of five cohorts. *Diabetologia* **55**, 1971-1977 (2012).
2. Zhou, K. *et al.* Common variants near ATM are associated with glycemic response to metformin in type 2 diabetes. *Nat Genet* **43**, 117-120 (2011).
3. Kooy, A. *et al.* Long-term effects of metformin on metabolism and microvascular and macrovascular disease in patients with type 2 diabetes mellitus. *Arch Intern Med* **169**, 616-625 (2009).
4. Tkac, I. *et al.* Pharmacogenomic association between a variant in SLC47A1 gene and therapeutic response to metformin in type 2 diabetes. *Diabetes Obes Metab* **15**, 189-191 (2013).
5. Choi, J.H. *et al.* A common 5'-UTR variant in MATE2-K is associated with poor response to metformin. *Clin Pharmacol Ther* **90**, 674-684 (2011).
6. Stocker, S.L. *et al.* The effect of novel promoter variants in MATE1 and MATE2 on the pharmacokinetics and pharmacodynamics of metformin. *Clin Pharmacol Ther* **93**, 186-194 (2013).
7. Goswami, S. *et al.* Genetic variants in transcription factors are associated with the pharmacokinetics and pharmacodynamics of metformin. *Clin Pharmacol Ther* **96**, 370-379 (2014).
8. Hoffmann, T.J. *et al.* Next generation genome-wide association tool: design and coverage of a high-throughput European-optimized SNP array. *Genomics* **98**, 79-89 (2011).
9. Banda, Y. *et al.* Characterizing Race/Ethnicity and Genetic Ancestry for 100,000 Subjects in the Genetic Epidemiology Research on Adult Health and Aging (GERA) Cohort. *Genetics* **200**, 1285-1295 (2015).
10. Kvale, M.N. *et al.* Genotyping Informatics and Quality Control for 100,000 Subjects in the Genetic Epidemiology Research on Adult Health and Aging (GERA) Cohort. *Genetics* **200**, 1051-1060 (2015).
11. Tarasova, L. *et al.* Association of genetic variation in the organic cation transporters OCT1, OCT2 and multidrug and toxin extrusion 1 transporter protein genes with the gastrointestinal side effects and lower BMI in metformin-treated type 2 diabetes patients. *Pharmacogenet Genomics* **22**, 659-666 (2012).
12. Becker, M.L., Visser, L.E., van Schaik, R.H., Hofman, A., Uitterlinden, A.G. & Stricker, B.H. Genetic variation in the multidrug and toxin extrusion 1 transporter protein influences the glucose-lowering effect of metformin in patients with diabetes: a preliminary study. *Diabetes* **58**, 745-749 (2009).
13. Dujic, T. *et al.* Organic cation transporter 1 variants and gastrointestinal side effects of metformin in patients with Type 2 diabetes. *Diabet Med* **33**, 511-514 (2016).
14. Christensen, M.M. *et al.* The pharmacogenetics of metformin and its impact on plasma metformin steady-state levels and glycosylated hemoglobin A1c. *Pharmacogenet Genomics* **21**, 837-850 (2011).

Supplementary Figure 1 Regional plots of the association of *SLC22A1/SLC22A2*, *SLC47A1/SLC47A2* and *SLC22A4* loci with metformin glycaemic response assessed as HbA_{1c} reduction - meta-analysis results of the two largest MetGen cohorts, the GoDARTS and PMT2-EU study. A total of 3,471 variants with MAF ≥ 1% were tested in 6,964 participants. None of the variants reached the significance threshold after correction for multiple testing (p >1.6 x 10^{-4}).

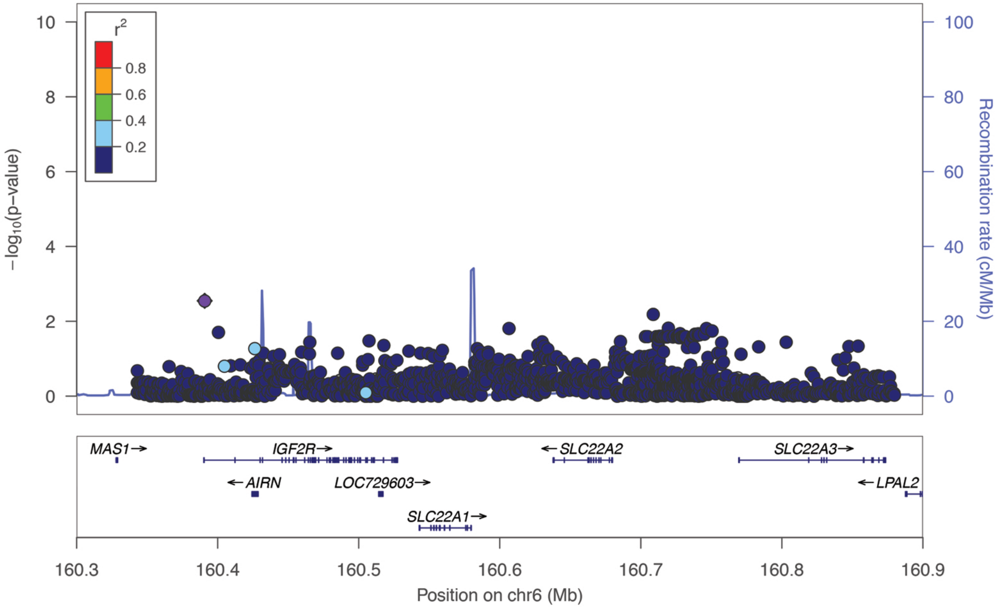

r2
0.8
0.6
0.4
0.2
−log10(p−value)
Recombination rate (cM/Mb)
MAS1→
IGF2R→
←SLC22A2
SLC22A3→
←AIRN
LOC729603→
←LPAL2
SLC22A1→
160.3
160.4
160.5
160.6
160.7
160.8
160.9
Position on chr6 (Mb)

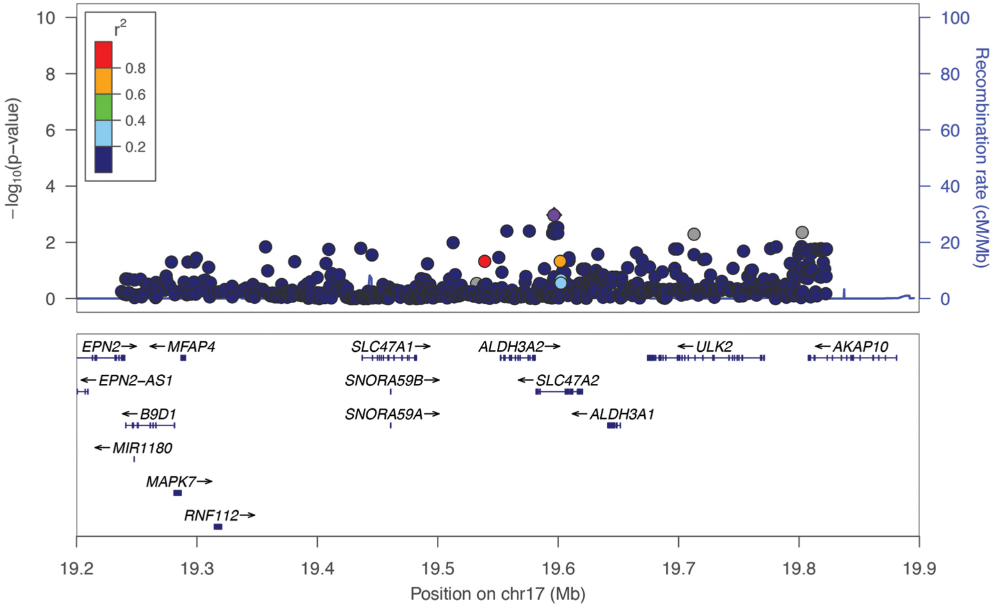

r2
0.8
0.6
0.4
0.2
−log10(p−value)
Recombination rate (cM/Mb)
EPN2→
←MFAP4
SLC47A1→
ALDH3A2→
←ULK2
←AKAP10
←EPN2-AS1
SNORA59B→
←SLC47A2
←B9D1
SNORA59A→
←ALDH3A1
←MIR1180
MAPK7→
RNF112→
19.2
19.3
19.4
19.5
19.6
19.7
19.8
19.9
Position on chr17 (Mb)

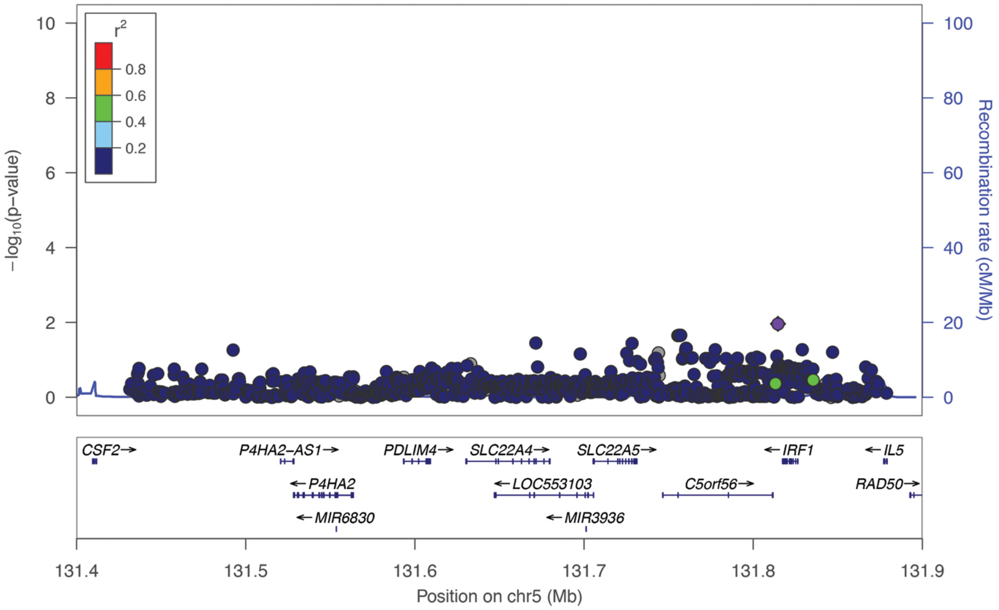

r2
0.8
0.6
0.4
0.2
−log10(p−value)
Recombination rate (cM/Mb)
CSF2→
P4HA2-AS1→
PDLIM4→
SLC22A4→
SLC22A5→
←IRF1
←IL5
←P4HA2
←LOC553103
C5orf56→
RAD50→
←MIR6830
←MIR3936
131.4
131.5
131.6
131.7
131.8
131.9
Position on chr5 (Mb)
